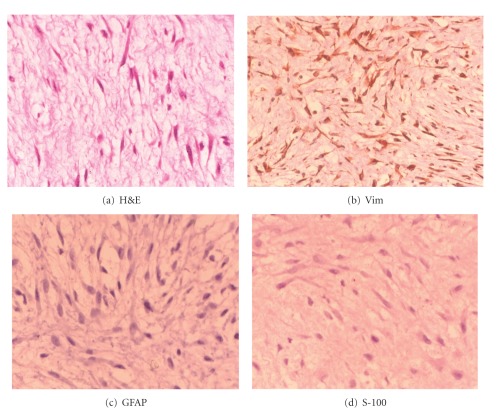# Periocular Myxoma in a Child

**DOI:** 10.1155/2012/739094

**Published:** 2012-04-29

**Authors:** Dolores Ríos y Valles-Valles, Ana María Vera-Torres, Héctor A. Rodríguez-Martínez, Abelardo A. Rodríguez-Reyes

**Affiliations:** ^1^Ophthalmic Pathology Service, Hospital “Dr. Luis Sánchez Bulnes”, Asociación Para Evitar la Ceguera en México, Vicente García Torres 46, 04030 San Lucas Coyoacán, DF, Mexico; ^2^Department of Oculoplastics and Orbit, Hospital “Dr. Luis Sánchez Bulnes”, Asociación Para Evitar la Ceguera en México, Vicente García Torres 46, 04030 San Lucas Coyoacán, DF, Mexico; ^3^Laboratorio de Investigaciones Anatomopatológicas “Roberto Ruiz Obregón”, Departamento de Medicina Experimental, Facultad de Medicina, UNAM y Hospital General de México, Dr. Balmis 148, 06726 Colonia Doctores, DF, Mexico

## Abstract

Myxomas are locally invasive, benign mesenchymal neoplasms with odontogenic, osteogenic, or soft tissue origin. Facial myxomas probably account for less than 0.5% of all paranasal sinus and nasal tumors. We report a case of a left painless periocular mass in a 11-month-old girl. The lesion was resected with a clinical diagnosis of lacrimal sac tumor. Histopathology and immunohistochemistry proved the tumor to be a myxoma. There has been no recurrence after 4 years of followup. Midfacial myxomas should be differentiated from other benign and malignant tumors such as dermoid, hamartoma, neurofibroma, nasolacrimal duct cyst, and sarcomas in particular embryonal rhabdomyosarcoma. Because of the infiltrative nature of these tumors, a wide surgery is required to achieve clear resection margins and avoid recurrence.

## 1. Introduction

Childhood myxomas of the head and neck are rare, but some well-documented cases have been published in the literature.

They may be related to dental malformations or missing teeth, but many occur without any such abnormalities.Their local aggressiveness and ability to erode bone should not be underestimated, thus surgical resection with free margins of these tumors is mandatory [1]. A case of periocular myxoma in a baby girl is described. 

## 2. Case Report

An otherwise healthy 11-month-old baby girl presented with an 8-day complaint of painless swelling in the left medial canthal region. Her family reported a mild upper respiratory infection treated with antibiotics 5 days earlier. No accompanying history of preceding trauma, nasal, ocular, or dental disease was mentioned. 

**Figure 1:**
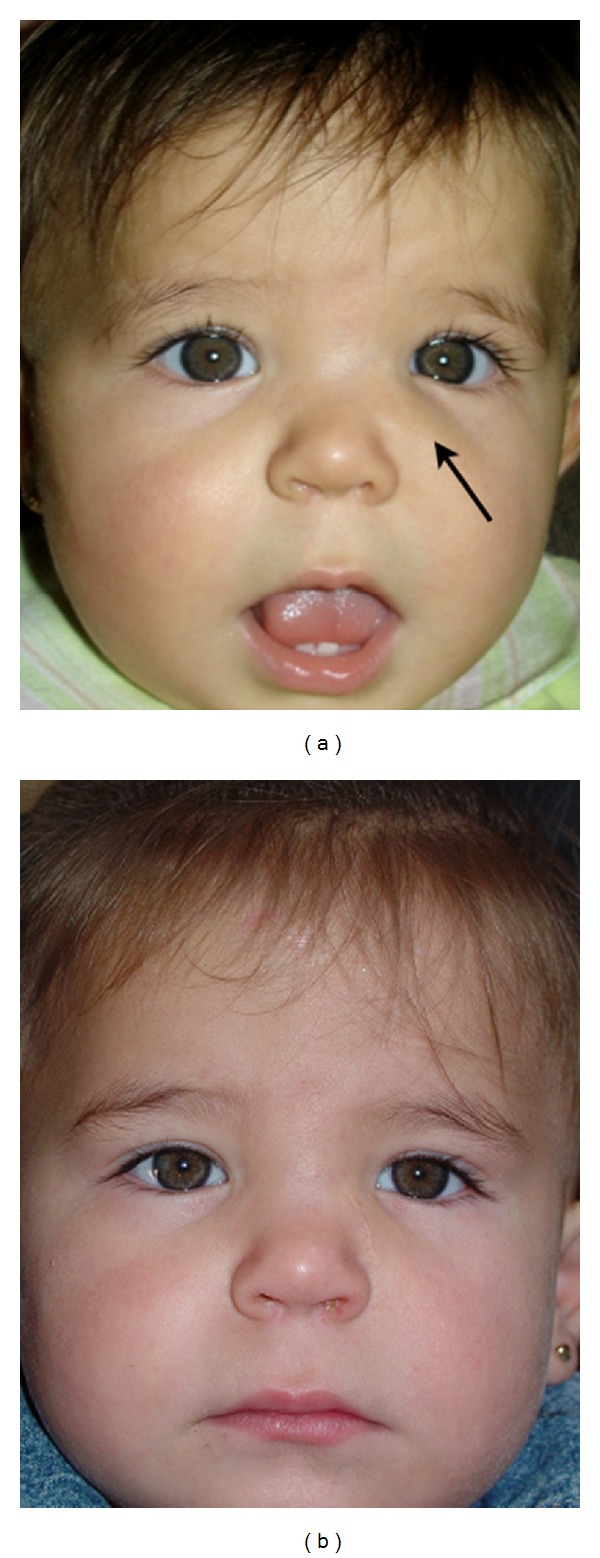


Physical examination of the child revealed a nontender, firm, and fixed infraorbital mass on the left side that appeared to be arising from the maxillary sinus. The margins of the swelling were ill-defined and measured 2 × 2 cm. The skin overlying the lesion was intact. Ophthalmological examination confirmed no direct involvement of her left eye and lacrimal pathway (Figure 1(a)).

**Figure 2:**
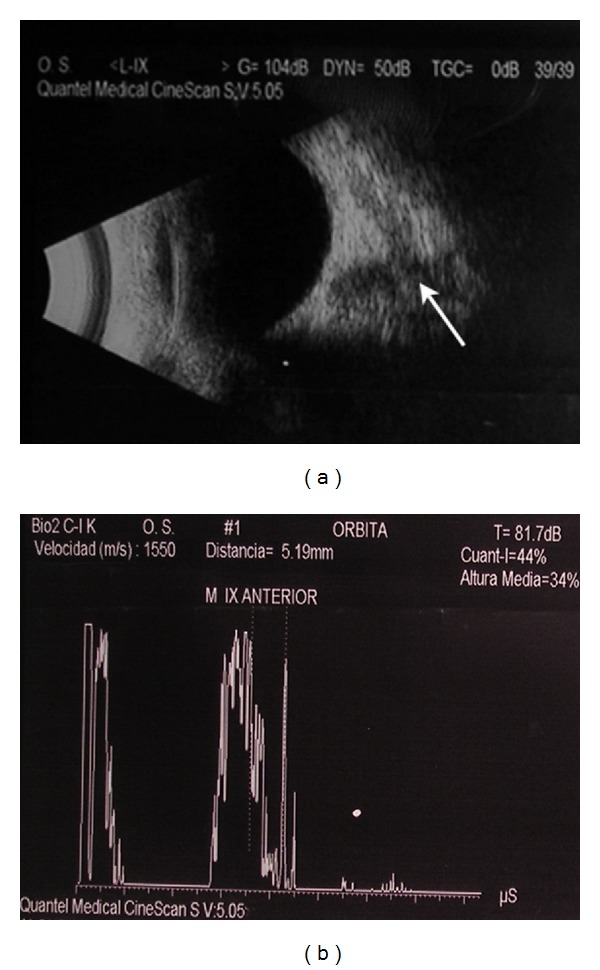


**Figure 3:**
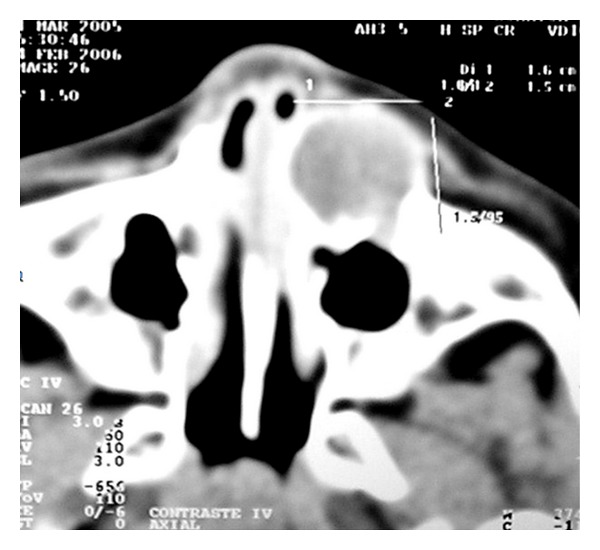


Echographic examination of the left orbit disclosed an ill-demarcated mass with irregular internal structure and medium internal reflectivity (44%). Peripheral bony destruction was noted (Figure 2). CT scan demonstrated an heterogeneous mass affecting the anterior aspect of the maxillary sinus with a possible clinical diagnosis of lacrimal sac tumor (Figure 3).

**Figure 4:**
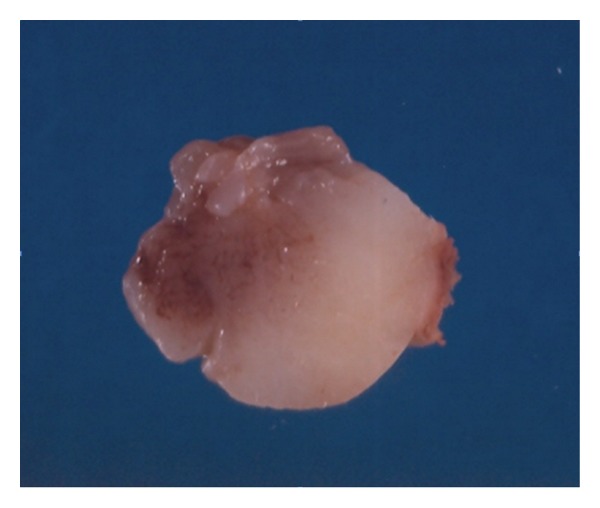


**Figure 5:**
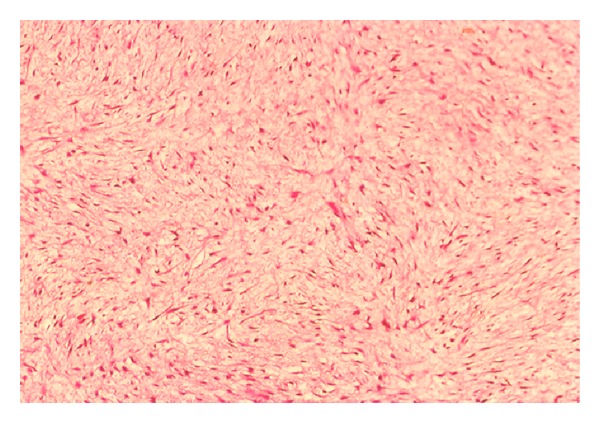


**Figure 6:**
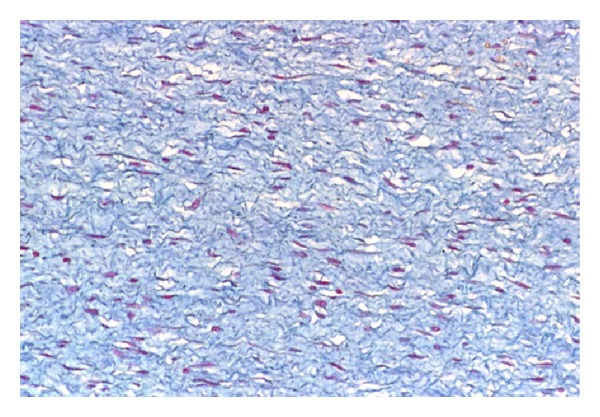


Excisional biopsy of the lesion was performed and the specimen submitted to pathology. Gross examination revealed a well-circumscribed nonencapsulated solid tumor measuring 26 mm on its largest diameter. The tumor was soft in consistency. The cut surface had a glistening gray-yellowish appearance (Figure 4). Histopathological examination disclosed a loose fibromyxoid stroma with sparse stellate and spindle cells. The cells had small hyperchromatic nuclei, fine chromatin, and basophilic stellate cytoplasm. There was no cellular pleomorphism or nuclear atypia. No obvious mitotic figures or necrotic areas were observed (Figure 5). Special stain for mucin by alcian blue was strongly positive in the loose stroma confirming the acid mucopolysaccharide nature of the tumor (Figure 6). Immunohistochemically, the cytoplasm of the stellate and spindle cells was strongly positive for vimentin and negative for desmin, S-100 and GFAP (Figure 7). The histopathological and immunohistochemical features were consistent with the diagnosis of periocular myxoma, and ruled out other tumors such as embryonal rhabdomyosarcoma, neurofibroma, and glioma, respectively.

After the surgery, the patient returned for follow-up examination 7 days, 6 months, one year, and 4 years later. The wound healed without complication, and there has been no evidence of recurrence (Figure 1(b)).

## 3. Discussion

Stout, in 1948, provided the first extensive review and clear diagnostic criteria for myxoma. He defined myxomas as true neoplasms of primitive mesenchyma that do not metastasize [2].

Facial myxomas are benign slow-growing expansible tumors with odontogenic, osteogenic, or soft tissue origin [3]. They are generally seen in adolescents and adults and more frequently in the mandible and maxilla often associated with missing or impacted teeth [4, 5]. They probably account for less than 0.5% of all paranasal sinus and nasal tumors [1]. Myxoma is a rare tumor in childhood with similar sex incidence [6–10]. As in our patient they are painless masses that remain undetected for several months. When symptoms of nasal congestion, epistaxis, or face distortion occur, these lesions commonly have bony erosion [10]. The gross appearance of these tumors appears as smooth, rubbery, and gray-white spherical masses with gelatinous cut surface. The consistency may vary depending on the amount of fibrous tissue. They can simulate encapsulated lesions owing to the compression and condensation of the surrounding tissues but lack a true capsule and are locally infiltrative. The typical histologic appearance is of a hypocellular tumor composed of undifferentiated spindle and stellate cells, arranged in a loose mucoid stroma rich in hyaluronidase [11, 12]. Myxoma is to be distinguished from a spectrum of reactive and neoplastic lesions that may show prominent myxoid degeneration including nodular fasciitis, schwannomas, neurofibromas, myxoid chondrosarcoma, myxoid liposarcoma, and embryonal rhabdomyosarcoma. Unlike benign myxomas, these sarcomas display areas of increased cellularity, pleomorphism, mitotic activity, and a rich vascular network [12]. Myxoma is unresponsive to chemotherapy and is poorly responsive to radiotherapy [10]. Surgery remains the treatment of choice [1, 10]. However, because of the unencapsulated and infiltrative nature of these tumors, extensive surgery is often required to achieve clear resection margins and avoid recurrence. A review of reported cases demonstrated that patients who underwent a complete resection fared well with no recurrence in the short-term follow-up period [10].

In conclusion myxoma should be considered as a differential diagnosis in children with a painless periocular mass and differentiated from more malignant tumors, such as embryonal rhabdomyosarcoma. The immunohistochemistry is a useful complementary tool to confirm the diagnosis of myxoma and to rule out other benign and malignant myxoid tumors. Complete resection of the tumor with free margins is the treatment of choice.

## Figures and Tables

**Figure 7:**